# Mental health status of Chinese nursing students during night shifts in clinical placements: a cross-sectional analysis

**DOI:** 10.3389/fpsyg.2025.1655437

**Published:** 2026-01-13

**Authors:** Hongmei Wang, Jiafang Xiao, Taotao Zhang, Violeta Lopez

**Affiliations:** 1 School of Nursing, Hubei University of Medicine, Shiyan, China; 2 School of Nursing and Allied Medical Sciences, Holy Angel University, Angeles, Philippines; 3 Academic Department, International Institute of Health Sciences, Welisara, Sri Lanka

**Keywords:** Chinese, clinical practice, night shift, nursing students, psychological symptoms

## Abstract

**Background:**

Nursing students must complete the number of hours spent in clinical practice at various shift to bridge the gap between theory and practice. However, studies found that night shifts had significant impact on the mental health of nurses as they suffer from insufficient sleep and poor sleeping quality. There is, however, little is known about the mental health of undergraduate nursing students working night shifts during clinical practice.

**Aim:**

This study assessed nursing students’ mental health during night-shift clinical practice and compared their outcomes to the Chinese national norm.

**Methods:**

A cross-sectional study of 203 nursing students from three Shiyan hospitals completed the Symptom Check-list 90 (SCL-90) online questionnaire. The study was conducted in March 2024. Data were analyzed via SPSS 26.0 with descriptive statistics, independent t-tests, and Spearman correlation.

**Results:**

Students worked 1 to 2 night shifts weekly; their total mean SCL-90 score (2.10 ± 0.71) exceeded the national norm (1.44 ± 0.43), with significant differences across all SCL-90 dimensions (*p* < 0.05). Night-shift frequency correlated positively with psychological symptoms.

**Conclusion:**

Nursing students on night shifts showed elevated SCL-90 scores across all dimensions. Nursing schools and hospitals should recognize this impact, develop interventions, optimize shift allocation, monitor mental wellbeing, provide enhanced preceptorship, and re-evaluate the necessity of night-shift clinical practice for competency development.

## Introduction

Rotating shifts are universal standard in nursing education designed to provide round-the-clock care for patients. There has been growing recognition among nursing authorities regarding the unique properties of night shift, which are essential for nursing students to acquire necessary competencies (Brown and Sandiford, 2022). The unique features of night shifts include lower light and noise levels allowing student nurses time to conduct collaborative and high-quality care. These shifts enable students to study articles, guidelines, and patient medical records (Nilsson et al., 2008; Powell, 2013). Night shifts also provide different experiential learning landscape for nursing students and countries such as Croatia, UK, Italy, Spain, and the United States have implemented night shifts clinical placements (Dobrowolska et al., 2015).

However, night shift can disrupt nursing students’ sleep patterns leading to fatigue, disillusionment, negative work attitudes, and burnout (James et al., 2020). In addition, night shift nurses showed poorer mental health than non-night shift nurses (Roman et al., 2023). In contrast to experienced nurses, nursing students are typically introduced to night shift work for the first time during clinical placement; therefore, findings from nurses may not be generalize to nursing students (Postma et al., 2017).

In China, undergraduate nursing students are assigned to registered nurse preceptors in hospitals under a “preceptor-shadowing” model, including night shifts to fulfill clinical learning objectives. This requirement has been a longstanding component of the Chinese national nursing curriculum since the re-establishment and standardization of modern higher nursing education in the 1980s. Night-shift training typically takes place during the final year clinical placement. Nursing students with documented mental health or other significant medical conditions may apply for exemptions in principle (Ministry of Education of the People's Republic of China, 2018). A typical night shift lasts 8 h, for example, from 12:00 a.m. to 8:00 a.m. the following day. This practice is standard across the vast majority of nursing programs in China, guided by the National Standards for Nursing Education, with slight variations in the required number of night shifts among institutions (Xiong, 2022; Zhou and Fan, 2016).

From an international perspective, nursing students in European countries such as the Czech Republic, Italy, Poland, Portugal, and Slovakia also complete night-shift rotations to gain a comprehensive understanding of 24-h patient care (Dobrowolska et al., 2020). However, their research on the effects of night shifts primarily focuses on the learning experience of student nurses. Some studies indicate that nursing students on night shifts reported lower job satisfaction, higher fatigue, and inferior clinical learning compared to other nursing students on day shifts (Belingheri et al., 2020; Roman et al., 2023), there is limited research on the impact of night shifts on the mental health of nursing students.

In the process of transforming student nurses into registered nurses, they inevitably need to undertake such clinical schedules in China. The Chinese National Nursing Development Plan highlights the significance of strengthening the nurse team and promoting high-quality nursing development. Nursing students are considered an important resource for future nursing workforce and thus must have good physical and mental health (National Health Commission of the People’s Republic of China, 2022). While the impact of night shifts on nurses’ mental health is well-documented, little research has focused on undergraduate nursing students who complete night shifts during clinical training in China. Therefore, our study aimed to examine the mental health status of nursing students working night shifts. We also aimed to explore targeted interventions regarding their mental well-being, promoting the smooth transition from student nurses to registered clinical nurses.

## Methods

### Design

The study employed a cross-sectional study design. It is descriptive research that involves analyzing the information about a population at a specific point in time and also can be used to determine relationships between variables (Wang and Cheng, 2020). This method may limit generalizability due to potential sampling bias, as participants were not randomly selected from the broader population of nursing students on night shift internships.

### Setting and participants

In our study, convenience sampling method was used to recruit student nurses. The inclusion criteria were student nurses who have undergone a 40-week clinical practice from June 2023 to March 2024 in three university-affiliated Grade A general hospitals located in Shiyan City, Hubei Province, China. Grade A hospitals are classified as the highest hospital grade in China. The exclusion criteria are student nurses are those suffering from anxiety, depression, obsessive-compulsive disorder, and other mental disorders before clinical practice. Pre-existing mental disorders were medically confirmed by the mental health teacher in our research team. Student nurses who are suspended from clinical practice for over one month due to medical or personal reasons were also excluded. Initially, 210 students met the inclusion criteria and consented to participate in the study. Later, four students withdrew from the study due to illness or personal leave, and three students failed to complete the questionnaire. The final sample was 203 (96.7%). To assess potential bias, we compared the key demographic characteristics (e.g., age, gender, duration of clinical practice) of participants and non-participants. No significant differences were found between the two groups (*p* > 0.05), suggesting that sample was broadly representative and no response bias. Furthermore, the study’s aims and the voluntary nature of participation were clearly stated in the consent process to mitigate this concern.

### Outcome measures

We collected demographic information on participants’ age, gender, duration of clinical practice, and the frequency of their night shifts. Participants completed the tool independently without researcher supervision. We used the Chinese version of the Symptom Check-list 90 (SCL-90). The original SCL-90 was developed by Leonard Derogatis in mid-1970s, also known as Self-Reporting Inventory, and is considered one of the world’s most famous mental health test scales. SCL-90 consists of nine symptom dimensions including somatization, obsessive-compulsive, interpersonal sensitivity, depression, anxiety, hostility, phobic anxiety, paranoid ideation, and sychoticism, all of which reflect mental health conditions (Lanyon, 2007; Derogatis et al., 1973). The scale utilizes a 5-point Likert scale to assess the nine symptom factors from 1 (absent) to 5 (severe). A higher score for a symptom factor suggests a more pronounced issue related to the corresponding psychological symptoms. The higher the total score, the lower the overall level of mental health. Since the 1990s, Chinese researchers have utilized the SCL-90 to investigate and analyze the mental health of nurses. We also used the SCL-90 to compare the mental health status of our sample with the Chinese national norms obtained from a large-scale testing of the SCL-90 in the normal population in China (Wang, 1984).

In our study, the SCL-90 demonstrated good internal consistency in our study, with a total scale Cronbach’s alpha of 0.83. Subscale Cronbach’s alphas were as follows: Somatization (0.82), Obsessive-Compulsive (0.85), Interpersonal Sensitivity (0.84), Depression (0.88), Anxiety (0.83), Hostility (0.79), Phobic Anxiety (0.77), Paranoid Ideation (0.78), and Psychoticism (0.76). These values are consistent with the tool’s established reliability in Chinese populations (Song et al., 2020), further confirming its suitability for our sample.

### Data collection

Data collection was carried out over a four-week period beginning in March 2024 using the Symptom Checklist-90 (SCL-90) administered through an online questionnaire platform. The SCL-90 is a widely used self-report tool designed to assess psychological distress across nine symptom dimensions. Ethical approval for the study was obtained from the Science and Technology Ethics Committee of Hubei University of Medicine (Reference Number: 2024-RE-019). Participants received a written information sheet outlining the study purpose, procedures, and their rights, including voluntary participation and the option to withdraw at any time without penalty. Informed consent was implied upon the submission of the completed online questionnaire, which was distributed via an anonymized mobile survey link. To ensure confidentiality, no personal identifiers or IP addresses were collected, and all data were stored in a password-protected repository accessible only to the research team. Data were reviewed for completeness before analysis, and security measures were implemented to maintain anonymity, integrity, and secure handling of all responses.

### Data analysis

Survey data were entered into Statistical Package for Social Science (SPSS) version 26.0. Descriptive statistics (mean, standard deviation) were calculated. Independent t-test was used to examine difference in mental health status between our sample and Chinese mental health national norms. The normality of the data was assessed prior to the independent t-test. The results indicated that all key variables (night shift frequency and SCL-90 total/subscale scores) followed a normal distribution (Shapiro–Wilk *W* = 0.97–0.99, *p* > 0.05), supporting the use of parametric statistical methods. Spearman correlation was used to determine the association between SCL-90 and frequency of night shifts.

## Results

### Participant characteristics

A total of 210 undergraduate nursing students consented to participate in this study but only 203 (96.7%) questionnaires were valid for final data analysis. The mean age was 22 ± 1.54 years, the duration of their clinical practice was 8.25 ± 1.15 months, and the frequency of night shift was 1–2 nights a week. Demographic characteristics (e.g., age, gender, duration of clinical practice) of nursing students showed no statistically significant differences (*p* > 0.05). The results indicated that while male students had slightly lower scores than females on the obsessive-compulsive, depression, anxiety, and psychoticism factors, but no statistically significant differences.

### Comparison between SCL-90 scores of night shift nursing students and Chinese mental health national norms

Nursing students who work night shifts exhibited elevated SCL-90 scores across all dimensions, surpassing the national average for China, reporting moderate symptom distress. The results of the t*-*test showed statistically significant differences in the symptomatology factor scores of somatization, obsessive-compulsive symptoms, interpersonal sensitivity, depression, anxiety, hostility, phobic anxiety, paranoid ideation and psychoticism (*p* < 0.05). The levels of somatization, obsessive-compulsive symptoms, interpersonal sensitivity, depression, anxiety, hostility, phobic anxiety, paranoid ideation, and psychoticism among nursing students working night shifts showed significant severity compared to the national average of Chinese mental health national norms. The details are shown in Table 1.

**Table 1:** Comparison between SCL-90 scores of night shift nursing students and Chinese mental health national norms.

Aspects	Night shift nursing students (*n* = 203) Mean (SD)	Chinese national norms (*n* = 1,388) Mean (SD)	*t*	*P*
Somatization	2.13 ± 0.69	1.37 ± 0.48	15.621	0.001
Obsessive-compulsive symptoms	2.28 ± 0.70	1.62 ± 0.58	13.466	0.001
Interpersonal sensitivity	2.09 ± 0.76	1.65 ± 0.51	8.228	0.001
Depression	2.13 ± 0.76	1.50 ± 0.59	11.668	0.001
Anxiety	2.12 ± 0.74	1.39 ± 0.43	14.074	0.001
Hostility	2.06 ± 0.81	1.49 ± 0.56	10.297	0.001
Phobic anxiety	1.95 ± 0.80	1.23 ± 0.41	12.852	0.001
Paranoid ideation	2.00 ± 0.79	1.43 ± 0.57	10.226	0.001
Psychoticism	1.97 ± 0.79	1.29 ± 0.42	12.162	0.001
Total score	2.10 ± 0.71	1.44 ± 0.43	13.202	0.001

### Correlation analysis of nursing students’ night shift frequency and SCL-90 factors

The correlation between night shift frequency and SCL-90 total scores was positively correlated [*r* = 0.335, *p* < 0.01, 95% CI (0.16, 0.46)]. Correlation analysis demonstrated significant positive correlations (*p* < 0.01) of night shift frequency with somatization (0.281), obsessive––compulsive symptoms, interpersonal sensitivity, depression, anxiety, hostility, phobic anxiety, paranoid ideation and psychoticism (*p* < 0.05). The levels of somatization, obsessive-compulsive symptoms, interpersonal sensitivity, depression, anxiety, hostility, phobic anxiety, paranoid ideation, and psychoticism among nursing students working night shifts showed significant severity compared to the national average of Chinese mental health national norms. The details are shown in Table 2.

**Table 2:** Correlation analysis between night shift frequency of nursing students and SCL-90 factors.

Aspects	Frequency of night shift	Somatization	Obsessive-Compulsive symptoms	Interpersonal sensitivity	Depression	Anxiety	Hostility	Phobic anxiety	Paranoid ideation	Psychoticism	Other	Total score
Frequency of night shift	1	0.281**	0.286**	0.335**	0.331**	0.364**	0.308**	0.335**	0.294**	0.342**	0.316**	0.335**
Significance		0.000	0.000	0.000	0.000	0.000	0.000	0.000	0.000	0.000	0.000	0.000
*n*	203	203	203	203	203	203	203	203	203	203	203	203

**At 0.01 level (double-tailed), Spearman correlation was significant.

## Discussion

Our study revealed that the SCL-90 scores of 203 nursing students working night shifts experience psychological symptomatology factors including somatization, obsessive-compulsive symptoms, interpersonal sensitivity, depression, anxiety, hostility, phobic anxiety, paranoid ideation, and psychoticism which is significantly different from Chinese mental health national norms scores (Wang, 1984). The factor scores were 0.44 to 0.76 points higher than the national norm, representing a relative increase of 26.7 to 58.5%. The factors with the greatest differences: paranoid Ideation (0.72 points higher, 58.5% relative increase), somatization (0.76 points higher, 55.5% relative increase), psychoticism (0.68 points higher, 52.7% relative increase), anxiety (0.73 points higher, 52.5% relative increase), The factor with the smallest difference: interpersonal sensitivity (0.44 points higher, 26.7% relative increase). The *p*-values for all comparisons were 0.000, indicating these differences are highly statistically significant. This result concurred with the findings of other studies that found compulsion, depression and anxiety among nursing students (Bahramirad et al., 2020). The poor mental health of nursing students in our study could be explained due to their limited exposure to clinical night shifts and insufficient confidence in their theoretical knowledge and nursing skills Moreover, the complex and variable nature of the night shift clinical environment could pose uncertainty surrounding various problems arising during the shift (Shen et al., 2025). As part of their practical experience, nursing students are expected to practice clinical skills and demonstrate theoretical knowledge during night shift. In this scenario, nursing students may experience heightened learning and practicing pressures which can exacerbate psychological stress, particularly among those working night shifts (Facioli et al., 2020; Karaca et al., 2019).

Our study found significant positive correlation between the frequency of night shifts and a range of psychological symptoms, including somatization, obsessive-compulsive symptoms, interpersonal sensitivity, depression, anxiety, hostility, phobic anxiety, paranoid ideation, and psychoticism (*p* < 0.01). The increased frequency of night shifts is associated with more severe mental health issues, as evidenced by higher rates of depression and anxiety among night shift workers (James et al., 2020). The results may be explained because night shift work disrupts the natural sleep patterns of nursing students, with frequent shifts further disrupting their circadian rhythm disruption (Boivin et al., 2022). Moreover, fatigue intensity adversely affects their mental wellbeing. Nursing students who frequently work night shifts for an extended period will experience disturbed sleep patterns. Their daytime activities will also be affected as their participation in social activities will diminish. Studies have shown that disruption in daytime activities may lead to a loss of their social role and a decrease in their sense of identity and belonging (Zhang et al., 2023). As a result, nursing students’ psychological state will be affected and they tend to have unhealthy psychological outcomes (Belingheri et al., 2022). These findings underscore the urgent need for nursing education institutions to develop targeted interventions—such as flexible night-shift scheduling and mental health support programs—to mitigate the adverse psychological impacts of frequent night shifts on nursing students.

### Strengths and limitations

This is the first study that assessed the impact of working night shifts on mental health of undergraduate nursing students as part of the clinical placement experiences. However, our study have some limitations. First, the study employed a cross-sectional study and convenience sampling in one geographic region of China. This method may limit generalizability due to regional sample and also cannot establish a true cause-and-effect relationship. Second, when using self-report data, nursing students may have underreported psychological distress due to social desirability bias or over-reported symptoms to seek support, which could introduce inaccuracies in the data. Third, athough the study provided us some understanding of the mental health status of nursing students, more research with increased sample size and additional survey questionnaires (e.g., resilience) are needed to better correlate with the SCL-90. A mixed-methods design can be used in future research to explore the strategies to support nursing students’ psychological wellbeing on shift duties.

## Implications

### Implications for clinical practice

The correlation between night shift frequency and the decline in psychological well-being among Chinese nursing students underscores the need for changes in clinical practice. Hospitals and healthcare institutions should implement structured scheduling policies that prioritize the mental health of student nurses. This includes adhering to a recommended minimum interval of 3–4 days between night shifts and limiting weekly night shift duration to no more than 8 h, as supported by evidence indicating that such measures help preserve sleep quality and reduce circadian rhythm disruption (Wyse et al., 2017). In addition, clinical supervisors and hospital staff should receive training to recognize signs of mental fatigue and emotional distress among nursing students (Postma et al., 2017). Continuous, on-site psychological support services should also be made accessible to help students process stress and avoid emotional burnout (Segura, 2024).

### Implications for future research

Further research is necessary to explore mediating and moderating factors. Of nights shifts among nursing students. Future studies to investigate how individual differences—such as coping style, resilience, or social support—affect how nursing students respond to night shift demands. Longitudinal research tracking students from the academic setting into early career stages could also provide insights into the long-term psychological effects of night shifts during training. Additionally, intervention-based studies are needed to test the efficacy of various support mechanisms—such as peer mentoring programs, mindfulness training, or flexible scheduling—in reducing negative mental health outcomes. Such research will help establish evidence-based guidelines that are specifically tailored to the Chinese nursing education context.

### Implications for nursing education

Nursing education programs should proactively equip students with skills to manage the psychological demands of night shifts. Integrating mental health literacy and self-regulation strategies into the curriculum, training in positive psychological techniques can empower students to recognize, accept, and adapt to emotional challenges (Dobrowolska et al., 2020). Educators and clinical teachers should also maintain open, consistent communication with students throughout their clinical placements, offering timely guidance and emotional support can help students navigate difficulties encountered during night shifts, in line with recommendations from the World Health Organization (2022). Ultimately, a well-rounded education that emphasizes both clinical competence and psychological preparedness is essential for sustaining the mental well-being of future nurses.

## Conclusion

This study examined the association between night-shift frequency and mental health outcomes among 203 Chinese undergraduate nursing students during mandatory clinical placements, providing insights into the psychological challenges specific to this population. Results related to this study underscore the need for targeted interventions to address physical discomfort and anxiety among nursing students in night-shift placements, such as flexible shift scheduling, pre-shift mental health briefings, and enhanced preceptor support during after-hours practice. These tailored strategies may help mitigate the observed distress while preserving the educational value of mandatory night-shift training.

## Data Availability

The original contributions presented in the study are included in the article/supplementary material, further inquiries can be directed to the corresponding author/s.
